# Potential role of insulin on the pathogenesis of depression

**DOI:** 10.1111/cpr.12806

**Published:** 2020-04-13

**Authors:** Xiao Han Zou, Li Hua Sun, Wei Yang, Bing Jin Li, Ran Ji Cui

**Affiliations:** ^1^ Jilin Provincial Key Laboratory on Molecular and Chemical Genetic The Second Hospital of Jilin University Changchun China

## Abstract

The regulation of insulin on depression and depression‐like behaviour has been widely reported. Insulin and activation of its receptor can promote learning and memory, affect the hypothalamic‐pituitary‐adrenal axis (HPA) balance, regulate the secretion of neurotrophic factors and neurotransmitters, interact with gastrointestinal microbiome, exert neuroprotective effects and have an impact on depression. However, the role of insulin on depression remains largely unclear. Therefore, in this review, we summarized the potential role of insulin on depression. It may provide new insight for clarifying role of insulin on the pathogenesis of depression.

## 1 INTRODUCTION

Diabetes is a metabolic disease characterized by hyperglycaemia due to the defect of insulin secretion or impaired insulin biological effect. A survey has shown that about 30% of people with diabetes also suffer from depression and 10% have severe depression, which suggests a close relationship between the two diseases.^1^, ^2^ The mechanism of depression as a complication of diabetes is not completely clear, but studies have found that insulin deficiency or insulin resistance is the symptom that can also be observed in major depression disorder.^3^, ^4^, ^5^

Insulin is the only hormone in the human body that reduces the blood glucose level and promotes the synthesis of glycogen, fat and protein. Several studies found that insulin had the ability to affect the nervous system.^6^, ^7^, ^8^ Insulin receptors were distributed in other parts of the brain, except for the parts related to food intake and energy.^9^, ^10^, ^11^ Experiments have gradually revealed other functions of insulin in the brain, for instance promoting memory, protecting neurons, regulating synaptic plasticity and maintaining HPA axis homeostasis.^12^, ^13^, ^14^ These effects of insulin may provide a background for its relationship with depression.^15^, ^16^

In this case, although there is no direct evidence supporting the link between insulin and treatment for depression, more and more researches support this hypothesis. Here, we review the relationship between insulin signalling and neurophysiological homeostasis, neurotrophic metabolism, cellular pathways and some survey statistics. The exploration of these links and the validation of more relevant mechanisms will develop a new impact on the way of the diagnosis and treatment of depression.

## 2 INSULIN AND DEPRESSION

As early as 1980s, many evidences proved that insulin influenced depression and functional insulin receptors were widely present in the brain.^17^, ^18^, ^19^ Data from clinical and epidemiological studies demonstrate a two‐way link between emotion and metabolic dysfunction. In young depression patients, insulin sensitivity is significantly decreased.^20^, ^21^ Compared with "low insulin" (1.5 mU/kg × min), "high insulin" (15 mU/kg × min) induces a more pronounced of the ability to remember word lists in human being.^22^ Intranasal delivery of insulin in awake mice can transmit regulatory and metabolic hormones across the blood‐brain barrier (BBB), significantly improve memory and downgrade anxiety levels in rats. Moreover, the insulin spray enters the nasal cavity through passages in the cribriform plate, thus olfactory bulb is the first region in the brain that receives insulin stimulation. Insulin does not cause damage to the olfactory neuroepithelium, nor does it affect the density and quantity of the olfactory nerve.^23^ In human experiments, the use of insulin for intranasal treatment of patients with impaired memory is benefit to their memory without altering their peripheral blood glucose and insulin levels.^24^, ^25^

In addition to the direct effect of insulin on the nervous system, in recent years, some findings suggested that the association was related to the reduction of insulin receptor or the activity of receptor (ie, insulin resistance) in brain,^26^, ^27^, ^28^ and the insulin resistance was positively associated with depression.^5^ Long‐term feeding of high‐fat diet induces peripheral insulin resistance. The CA1 hippocampus of the peripheral insulin resistance is taken out for extracellular recording, and it is found that neuronal insulin resistance occurred here. At the same time, the levels of the neuronal insulin receptor, insulin receptor substrate 1 (IRSs) and phosphorylation protein kinase B (Akt) are reduced, which in turn leads to neuronal stress (increased neurocortical hormone).^29^ Overnutrition and diabetes can selectively cause insulin resistance in different parts of the brain, disrupt homeostasis, affect the normal function of the brain and increase depression disease progression.^30^

**FIGURE 1:**
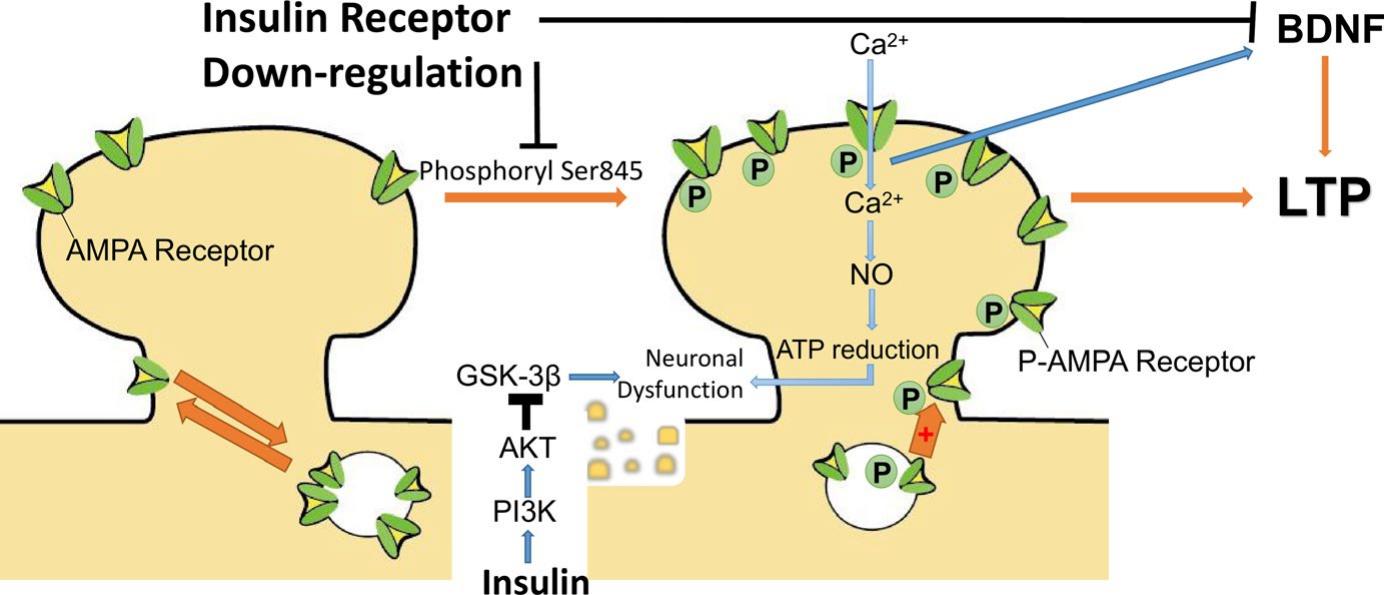
In general, phosphorylation of the 3‐hydroxy‐5‐methylisoxazole‐4‐propionic acid (AMPA) receptor Ser845 site and subsequent transfer of the receptor to the extrasynaptic site are the first steps in long‐term potentiation (LTP). Phosphorylated AMPA receptors cause CA^2+^ flow into nerve cells, which on the one hand leads to an increase of brain‐derived neurotrophic factor (BDNF) levels and exerts an antidepressant effect, on the other hand leads to an increase of NO levels, decreases ATP levels and impairs neuronal function. Downregulation of the insulin receptor results in downregulation of phosphorylation of Ser845 site and decreases receptors transport, as well as causes decrease in BDNF levels, all of which inhibit LTP. Insulin activates the phosphoinositide 3‐kinase (PI3K)/protein kinase B (AKT) pathway and inhibits the glycogen synthase kinase 3‐beta (GSK‐3β) pathway, thereby inhibiting neuronal dysfunction

In the hypothalamic insulin receptor expression downregulation rat model constructed using a lentiviral vector,^31^ phosphorylation of Ser845 (Serine 845 of 3‐hydroxy‐5‐methylisoxazole‐4‐propionic acid Receptor 1) was significantly reduced in rats. Phosphorylation of Ser845 is important for activity‐dependent trafficking of 3‐hydroxy‐5‐methylisoxazole‐4‐propionic acid (AMPA) receptor glutamate receptor 1 (GluR1) to extrasynaptic sites for subsequent delivery to synapses during long‐term potentiation (LTP) and is a "start" step of LTP by promoting expression of AMPA receptors at extrasynaptic sites. Insulin regulates surface transport and phosphorylation of AMPA receptors to enhance synaptic transmission in LTP and increasing the extrasynaptic pool of AMPA receptors resulted in stronger LTP.^32^, ^33^ At the same time, the activation of AMPA receptors leads to Ca^2+^ flood into the neurons, which mediates upregulation of mTOR and brain‐derived neurotrophic factor (BDNF) and produces antidepressant effects,^34^ on the other hand it subsequently produces NO. NO reduces intracellular ATP levels by inhibiting the mitochondrial respiratory chain, leading to neuronal dysfunction^35^ (Figure 1).

Selective downregulation of the hypothalamic insulin receptor was responsible for LTP deficiency in the CA1 region of the hippocampus, which also impaired synaptic plasticity in the hippocampus.^36^ At the same time, the levels of BDNF in hippocampus and amygdala were decreased (Figure 1). In behavioural experiments, the fixed time of Hypo‐IRAS rats increased significantly, the activity of forced swimming experiment decreased correspondingly, the sucrose intake of the sucrose preference test decreased, and the anxiety behaviour increased in the labyrinth test.^36^, ^37^ Overall, these data support the hypothesis that downregulation or inactivation of insulin receptors increases the risk of mood disorders. Insufficient insulin receptor signalling may be a contributing factor to the development of depression. After using insulin sensitizers to alleviate insulin resistance, the depressive behaviour of obese animals was alleviated.^38^, ^39^ However, in recent years, experiments have also shown that insulin receptor knockout has no effect on hedonic and anxiety behaviour in mice. The reason may due to the occurrence of compensatory.^40^

As the only hormone in the body that lowers blood glucose, in addition to its receptor, the hypoglycaemic effect of insulin is closely related to its influence on the nervous system. There have been experiments showing that major depression is associated with glucose/insulin metabolism.^41^ Studies have continued to monitor blood glucose levels. After continuous subcutaneous insulin injections to control blood glucose, blood glucose levels were found to be significantly related to the “tension” and “hedonic tone” scales. High blood glucose level has a negative impact on mood, and decreases the level of positive emotions and increases the level of negative emotions.^42^ Experiments have also shown that inducing low levels of blood glucose can benefit to human cognitive ability and allows them to remember more words.^43^

Results measured by seahorse XF extracellular flux analyzers extend the finding that abnormal glucose metabolism and abnormal mitochondrial function can be observed in patients with depression. High glucose (≥10 mmol/L) comparable to diabetic brain extracellular glucose level leads to inactivation of monophosphate‐activated protein kinase (AMPK), which causes neuronal mitochondrial dysfunction,^44^ and caused the hexokinase 1 (HK1) attached mitochondrial outer membrane (OMM) reduced HK1, an initial and rate‐limiting enzyme of glycolysis. Binding to OMM greatly enhances HK1’s own activity, couples cytosolic glycolysis to the function of mitochondrial oxidative phosphorylation and induces cells produce most of adenosine triphosphate (ATP). While powering the brain, it also prevents apoptosis and oxidative damage, protects neurons.^45^ In the case of abnormal mitochondrial function caused by hyperglycaemia, the protection of neurons by HK1 disappears.

Imbalances in brain glucose metabolism often occur in patients with depression.^46^ Glucose is the main energy source of brain cells, and its entry into brain cells is mediated by the glucose transporter (GLUT) family.^47^ In the human brain, glucose transporter 1 (GLUT1) and glucose transporter 3 (GLUT3) are mainly transmembrane glucose transporters. GLUT1 is mainly expressed in endothelial cells and astrocytes, which is essential for brain maturation and normal brain function.^48^Compared to healthy comparison subjects, DNA methylation of the core promoter regions of GLUT1 was significantly increased in depression patients’ brain cells, which reduced the efficiency of GLUT1 absorb glucose from blood vessels to cells, impaired brain metabolism. After treatment of depression patients, DNA methylation of GLUT1 promoter was significantly reduced. This may indicate that the successful treatment of depression is related to the increases of GLUT1.^49^ And insulin could increase the expression of GLUT1 in an AKT‐dependent manner and make GLUT have higher glucose transport activity.^50^, ^51^ Acute stimulation with insulin on the plasma membrane of cells increases phosphorylation of AKT protein and levels of GLUT1 protein. Pre‐treatment with AKT inhibitors can eliminate these effects. In addition, insulin stimulation did not affect GLUT12 protein levels on the plasma membrane of the cells.^51^

In addition to the effects of mitochondria on the nerves, the activation of the insulin‐induced phosphoinositide 3‐kinase (PI3K)/Akt signalling pathway increases cell viability (including nerve cells). Activation of Akt also results in the inactivation of glycogen synthase kinase 3‐beta (GSK‐3β). GSK‐3β regulates mitochondrial biogenesis, bioenergy, permeability and apoptosis. The use of GSK‐3β inhibitors as drugs may be in the treatment of neurological diseases. Inactivation of GSK‐3β prevents nerve cell death^52^, ^53^ (Figure 1).

Collectively, there have been many investigations about the effects of insulin and blood glucose on depression, but the results are not identical. Therefore, more research may be needed.

## 3 EFFECT OF INSULIN ON DEPRESSION

### 3.1 Insulin affects depression through hypothalamic‐pituitary‐adrenal axis

Under normal conditions, the negative feedback regulation of the hypothalamicpituitary‐adrenal axis (HPA) process is as follows: the corticotropin‐releasing hormone (CRH, also known as CRF) secreted by the hypothalamus activates the CRH1 receptor and induces the secretion of pituitary adrenocortical hormone (ACTH). Then, ACTH causes the adrenal gland to release glucocorticoids (cortisol in humans, corticosterone in rodents) (GC), which activate the glucocorticoid receptor (GR) and mineralocorticoid receptors(MR) to activates the negative feedback loop and restore homeostasis.^54^, ^55^ In major depression, the secretion of CRH is increased, and GC is overproduced, the sensitivity of GR is impaired, HPA negative feedback mechanism is damaged.^56^, ^57^ Studies have been conducted through dexamethasone/corticotrophin‐releasing hormone (DEX/CRH) test and insulin resistance evaluated by the homeostasis model assessment of insulin resistance (HOMA‐R) to demonstrate that HPA axis dysfunction is associated with insulin in patients with depression.^14^ Insulin has a certain degree of influence on hormones involved in HPA negative feedback.^58^

**FIGURE 2:**
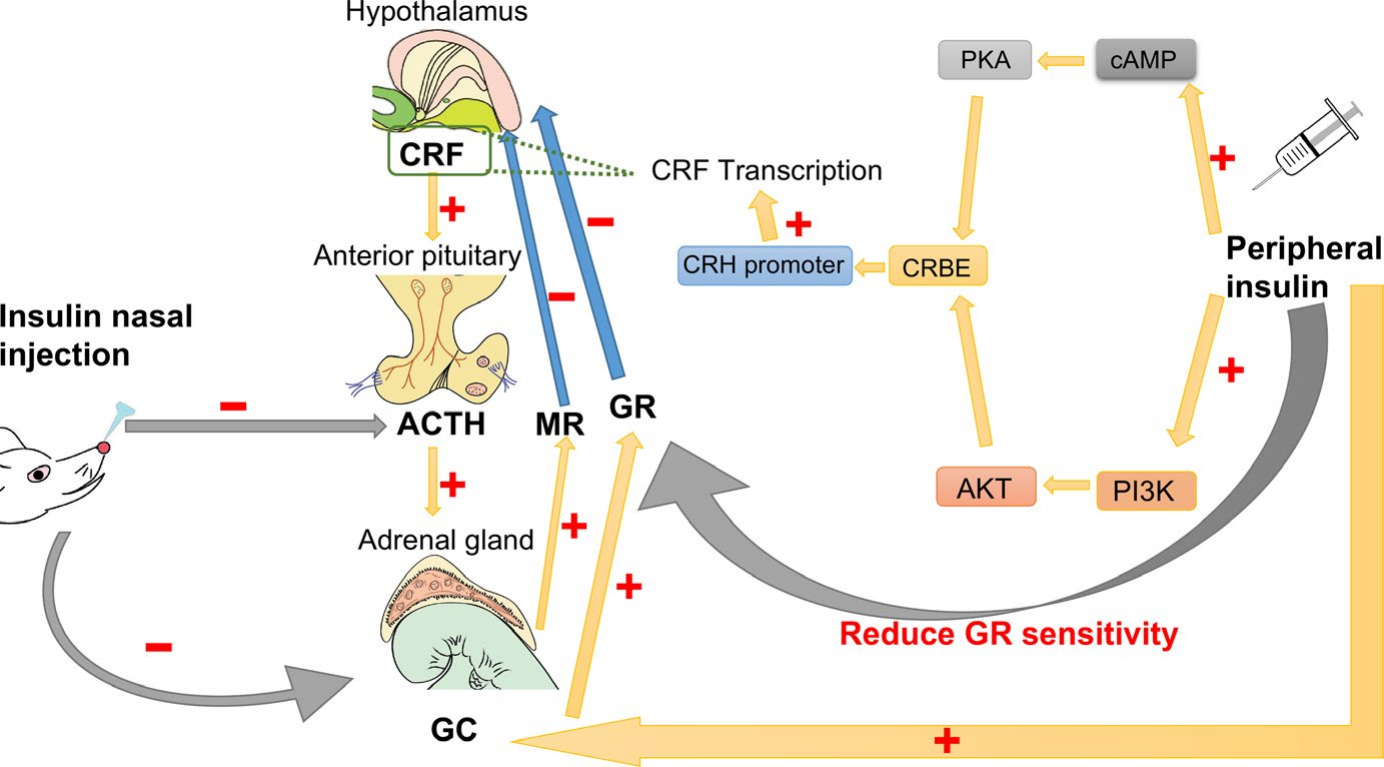
Peripheral injection of insulin can increase cyclic adenosine monophosphate (cAMP) response element‐binding protein (CREB) levels through cAMP/protein kinase A system AMP protein kinase A (PKA) and PI3K/AKT pathways, and then affects corticotropin‐releasing hormone (CRF) promoters, stimulates CRF gene transcription and increases CRF levels. At the same time, peripheral injection of insulin leads to an increase in glucocorticoids (GC) levels and a decrease in GC sensitivity. In contrast, insulin injection into the nasal cavity reduces adrenocortical hormone (ACTH) and GC levels

In an in vitro study of the mHypoA‐2 /12β hypothalamic cell line, insulin leads to activation of the CRH promoter primarily through activation of the cyclic adenosine monophosphate/AMP protein kinase A (cAMP/ PKA) pathway and the PI3K/ AKT pathway,^59^, ^60^ for CRH promoter contains cAMP response element‐binding protein (CREB) binding site that binds to the major transcription factors to stimulate transcription of the CRH gene.^61^, ^62^ After the use of PI3K inhibitors, insulin stimulation is reversed^60^ (Figure 2).

Phosphorylated Akt/CREB and BDNF and other downstream molecules in hippocampal neurons are downregulated when insulin resistance occurs.^63^, ^64^ In addition to affecting the CRF promoter, insulin can also affect the number of CRF neurons.^60^ Three hours after injecting insulin in vivo, the presence of CRH neurons increased.^65^ These evidences show the effect of excessive insulin on CRH may indicate its negative impact on depression.^60^, ^66^

CRF signals also play an important role in the physiological function of insulin. Rats exposed to chronic unpredictable mild stress (CUMS) have upregulated expression of the peptide urocortin 2 associated with corticotropin‐releasing factor, increased cAMP production, and cAMP/CREB pathway activates rats arcuate nucleus cells overexpressed factor signalling inhibitor 3 (SOCS3). Then, SOCS3 leads to insulin signalling involving the STAT3 and PI3K‐AKT‐FOXO1 pathways damaged by inhibiting the activation of insulin receptor substrate 2 (IRS2) and PI3K. Insulin signal returns to normal after using antidepressants.^67^

ACTH and GC levels increase after insulin injection at a rate of 1.5 mU/min/kg.^68^ Insulin can acutely enhance the effects of ACTH on androgen and glucocorticoid pathways.^69^ GC, a typical bi‐directional hormone that maintains normal physiological functions at normal concentrations, can cause nerve damage under high levels or chronic exposure.^70^ GC is also an important mediator of cognitive deficits caused by diabetes and impaired synaptic plasticity in the hippocampus. Elevated GC levels reduce insulin receptor signalling, including in the brain.^71^ The insulin signalling pathway mediates the symptoms of depression caused by GC disorders. Administration of exogenous GC can alter gene expression of insulin, leading to impaired learning and memory function.^72^ Glucocorticoid binds fully to MR and subsequently activates the c‐Jun N‐terminal kinase (JNK) in the hippocampus, which is associated with insulin receptor‐mediated inactivation of the Akt/ GSK3b pathway.^73^, ^74^ The Akt/ GSK3b pathway is the pathway required to induce long‐term potentiation and is the basis of learning and memory. The inactivation of this process leads to depressive symptoms such as decreased cell viability and cognitive deficits.^74^, ^75^ Insulin can also inhibit transient potassium currents by activating PI3K‐mediated signalling pathways. However, after GC pre‐treatment, insulin's effect on transient potassium channels disappears, leading to excitotoxicity and depression‐like behaviour in hippocampal neurons.^76^

After insulin is used to lower blood glucose, mRNA expression of hippocampal mineralocorticoid receptors, which is able to suppress HPA activity, is decreased. However, this condition is limited to normal animals. In animals with congenital hypoglycaemia, MR levels of mRNA remain at baseline levels after insulin administration. This may be because the animal's stress on insulin/glycaemia has disappeared. And not just for MR, in hypoglycaemic animals, insulin‐induced activation of GC is higher than normal. With repeated insulin stimulation, the levels of GC no longer changes significantly.^77^, ^78^ This may indicate that one of the mechanisms by which insulin affects the HPA axis is its effect on blood glucose. When insulin does not effectively lower blood glucose, its effect on the HPA axis is diminished. The effect of insulin on the FKBP5 gene also leads to a decrease in GR sensitivity (Figure 2). FKBP5‐encoded protein FKBP51 regulates GR function, reduces GC‐GR binding and GR sensitivity, promotes AKT dephosphorylation and downregulates AKT signalling. Overexpression FKBP51 can cause GR to be insensitive, and the GC level is too high.^79^, ^80^ Studies have shown that the expression of the FKBP5 gene is positively correlated with serum insulin and is associated with insulin resistance.^81^, ^82^ This process leads to prolonged activation of the HPA axis, which may be a risk factor associated with depression.

However, the effects on the HPA axis are different by insulin administration via the central or peripheral. Insulin single intranasal injection effectively lowers stress‐induced HPA axis responsiveness,^83^ reduces the levels of ACTH and GC levels and improves the memory and mood^84^ (Figure 2). Insulin is a benefit to the function of hippocampal and amygdala neurons, projecting directly or indirectly to the paraventricular nucleus and peritoneal region of the hypothalamus, inhibiting the HPA axis.^85^, ^86^ After 8 weeks of insulin administration via the nasal cavity, memory was improved and anxiety symptoms were alleviated.^25^ A decrease in the introversion and anxiety scores, as well as a decrease in depression and anger, confirms that intranasal insulin improves depression. This method does not involve the action of peripheral insulin.^70^, ^83^, ^84^ Interestingly, however, intranasal injection of insulin only reduces the weight of normal‐weight men, not the weight of obese men or women. Whether it is related to the sensitivity of insulin receptors in the case of sex hormones and obesity requires more experiments to explore.^84^, ^87^, ^88^

### 3.2 Insulin affects depression through neurotrophic effect

A growing evidence suggests that synaptic plasticity disorders and neuronal atrophy may be the causes of depression, and many antidepressants (such as ketamine) work by restoring synaptic plasticity and connectivity to key neural circuits.^89^ The mechanism by which insulin improves memory and alleviates depression may be that insulin regulates synaptic plasticity in the hippocampus.^90^, ^91^ It is generally believed that LTP and long‐term depression (LTD) are synaptic mechanisms of learning in mammalian brains. Changes in both LTD and LTP may represent changes in synaptic plasticity.^92^ In hippocampal CA1, insulin inhibits the transmission of excitatory synapses and promotes LTP.^93^ Insulin also regulates the endocytosis of AMPA receptors, which causes LTD of excitatory synaptic transmission.^90^, ^93^, ^94^ After reducing phosphorylation of the insulin receptor, the density of neuronal synapses decreased, the frequency of AMPA receptor minor excitatory post‐synaptic current (mEPSC) reduced and the plasticity of experience‐dependent dendritic arbour structural altered.^95^

**FIGURE 3:**
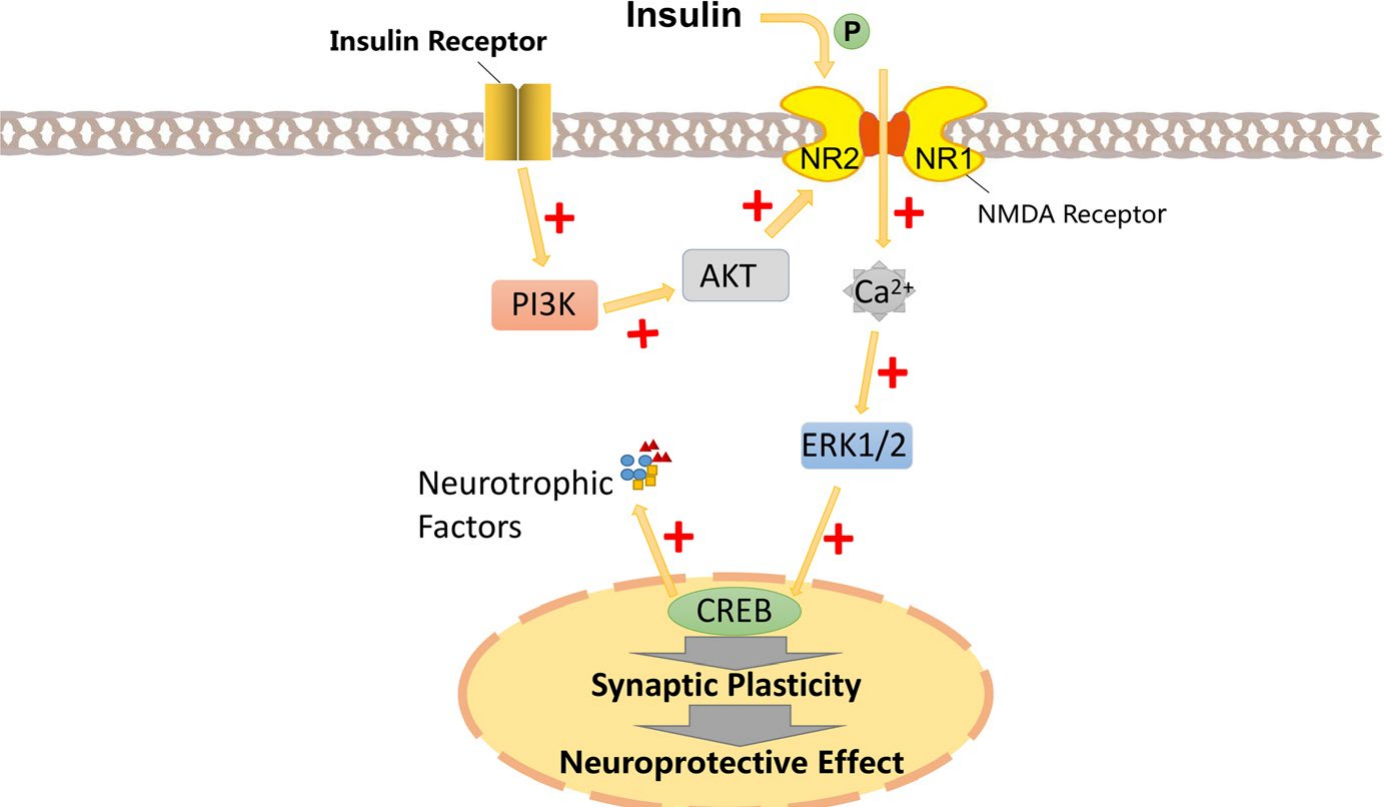
Insulin phosphorylates the N‐methyl‐D‐aspartic acid receptor (NMDA) receptor subunit NR2 and enhances NMDA receptor activity. Activation of the NMDA receptor causes Ca^2+^ to enter the cell, and Ca^2+^ induces nuclear signal transduction mediated by extracellular signal‐regulated kinase (ERK1/2) and then induces phosphorylation of CREB. At the same time, PI3K/AKT pathway, the downstream of the insulin receptor phosphorylates the NMDA receptor and then activates the CREB pathway to improve synaptic plasticity and promote learning and memory, produce neuroprotective effects, and produce neurotrophic factors

N‐methyl‐D‐aspartic acid (NMDA) not only plays an important physiological role in the development of the nervous system, such as regulating the survival of neurons, promoting the dendrites of neurons, the development of axon structure, but also important in restoring synaptic plasticity. Now, NMDA receptor is a important development direction of antidepressants.^96^ Insulin directly acts on the NMDA receptor, transiently phosphorylates the NMDA receptor subunit NR2 and enhances the current in the receptor to rapidly enhance NMDA receptor activity.^97^, ^98^ Activation of NMDA receptors leads to Ca^2+^ entry into cells, and Ca^2+^ induces extracellular signal‐regulated kinase (ERK1/2)‐mediated nuclear signal transduction and then induces CREB phosphorylation.^99^ At the same time, PI3K/AKT pathway in insulin downstream phosphorylates the NMDA receptor, then activates the CREB pathway to produce neurotrophic factors, improves synaptic plasticity, promotes learning and memory and produces neuroprotective effects.^100^ NMDA mediates insulin‐induced LTP and LTD, and this effect is specific. This process is inhibited after the use of NMDA receptor antagonists. The PI3K pathway plays an important role in this process, PI3K signalling enhances the current in the NMDA receptor, and inhibition of PI3K also prevents LTD.^98^, ^101^ After knocking out the insulin receptor, phosphorylation of the NMDA receptor subunit is reduced, and the downstream target PI3K and Akt are also reduced.^101^, ^102^ In chronic stress‐induced mouse models of depression (defined by reduced reward sensitivity), insulin receptor sensitizers restored their normal sugar‐water preference and increased subunit expression of NMDA receptors in the hippocampus.^103^ However, studies have also shown that injection of insulin into the lateral ventricle causes LTP deficiency, which indicates that local injection of insulin into the brain has an adverse effect on synaptic plasticity^104^ (Figure 3). Discrepancies between different experiments may be the difference of insulin concentration, the difference of measurement time, the difference of experiments conducted in vitro or in vivo, and so on. In conclusion, the role of insulin in synaptic plasticity requires more experiments to explore.

In addition to regulating neural plasticity, insulin plays a role in regulating neuronal growth, survival, proliferation and differentiation. There is a specific binding site for insulin in the neural crest origin, which suggests a correlation between insulin and neurite formation in human neuroblastoma SH‐SY5Y cells.^105^ The way it enhances neurite growth including stimulates DNA synthesis^106^ and increases the abundance of mRNA level of tubulin. Microtubules, cytoskeleton of axons and dendritic, the way insulin increases its mRNA is not covering all transcripts, but stabilized against degradation while increase the relative synthesis of tubulin. It has the same mechanism of how nerve growth factors act on microtubules and is common in the process of neurite elongation guided by neuritogenic polypeptides.^107^ The survival of hippocampal neural stem cells in vitro depends on insulin,^108^ and insulin receptor/PI3K/AKT pathway induces neural stem cell reactivation.^109^ The cessation of insulin causes autophagy‐dependent cell death, a kind of regulated cell death that requires autophagy mechanisms without the need for other cell death pathways.^110^ Some experiments have been carried out to explore the mechanism of this phenomenon. In insulin‐deficient hippocampal neural stem cells, GSK‐3β is an effector downstream of insulin, its inactivation reduces the level of autophagy‐dependent cell death, its activation leads to cell death.^111^ At the same time, the absence of insulin makes Ca^2+^ in the endoplasmic reticulum release, and elevated calcium levels activate AMPK.^110^, ^112^ (Figure 3). AMPK phosphorylates autophagy‐associated protein p62.^113^

Insulin is not only associated with autophagy‐dependent cell death, but also with autophagy in organelles. Autophagy refers to the production of alternative energy sources by lysosomal lysing organelles (such as mitochondria and endoplasmic reticulum) during body in hunger, and insulin inhibits the process through the mammalian target of rapamycin mTOR‐ or/and AKT pathway after ingestion of food.^114^ The more important role of autophagy regulated by energy and insulin is to clear the aging and damaged organelles.^115^ When autophagy is reduced, mitochondria‐derived oxidative stress increases and exacerbates insulin resistance.^116^, ^117^ In patients with insulin resistance, the mitochondrial DNA copy number is reduced.^118^, ^119^ In the streptozotocin model, which made by reducing insulin receptor phosphorylation and causes insulin resistance, adult hippocampal neural stem cells proliferate less, new neuronal formation is diminished, and GLUT and insulin‐like growth factor (IGF) levels reduced.^120^ The reduction of GLUT3 reduces the potential for neural stem cells to differentiate into neurons, as neonatal cells rely primarily on GLUT3 to transport glucose for energy.^121^ In this model, the content of reactive oxygen species (ROS) in hippocampal neural stem cells is greatly increased.^122^ Under normal circumstances, ROS mediates neuronal neurogenesis through PI3K/Akt signalling pathway. Whether in vivo or in vitro, reduced levels of ROS interfere with the function of normal NSCs and other progenitor cells.^123^ Content of endogenous ROS in neural stem cells is high, but excessive ROS may produce cytotoxicity leading to neuronal cell death, or inhibit proliferation of neural precursors^124^ (Figure 3).

Deficit of neurotrophic factors or disturbance of neurotrophic factor signalling pathways may be the primary cause of depression.^125^, ^126^ As a member of the neurotrophin protein family, decreased levels of BDNF lead to pathophysiological symptoms of depression.^127^ Normalized insulin signalling can both prevent and reverse BDNF transport defects.^128^ Insulin also increases BDNF mRNA levels and phosphorylation levels of Akt and GSK3b in a concentration‐dependent manner, thereby enhancing mouse memory in Y‐maze experiments and reducing depression‐like behaviour in forced swimming experiments.^129^ Experiment used streptozotocin, which is toxic to insulin‐producing B cells, to make a rat memory impairment model. Intranasal injection of insulin prevents STZ‐induced cholinergic dysfunction and mitochondrial function degradation, restoring BDNF and CREB expression,^130^ BDNF couple to CREB and is responsible for neurotrophic and neuroprotective role.^131^ In addition to affecting the BDNF/ CREB pathway, insulin also mediates the tropomyosin receptor kinase B (TrkB)/BDNF response and increases the expression of TrkB receptors and improves memory.^132^

The BDNF gene is located on chromosome 11P13 and is widely expressed in the brain. When codon 66 of the BDNF gene was mutated from valine to methionine (Val66Met), BDNF transmembrane transport and expression level decreased, which made BDNF an independent risk factor for senile depression. It was verified that the Met allele of BDNF was significantly correlated with depression.^133^, ^134^, ^135^ Met/ Met genotype of the BDNF has a higher probability of depression than others.^133^ BDNF Val/ Met and Met/ Met mutations are not only related to hippocampal volume and cognition, but also to obesity and type 2 diabetes.^136^ In high‐energy intake populations, Val/ Met is associated with increased insulin secretion and reduced prevalence of type 2 diabetes. People with Val/ Val alleles are more likely to develop diabetes than people with Val/ Met polymorphisms.^135^

### 3.3 Insulin affects depression through monoamine neurotransmitter

The hypothesis of monoamine neurotransmitter deficiency suggests that deficiency of monoamine neurotransmitters, namely norepinephrine and/or serotonin (5‐HT), underlies clinical depression. Classical antidepressants that increase central monoamine levels according to this mechanism are very useful for the treatment of depression.^137^

Monoamine 5‐HT is a recognized neurotransmitter associated with the pathophysiology of depression, and the deficiency of 5‐HT in certain areas of the brain can lead to depression. Tryptophan is converted to 5‐hydroxytryptophan (5‐HTP) under the catalysis of tryptophan hydroxylase (TPH), and 5‐HTP converts to 5‐HT by the catalysis of 5‐HTP decarboxylase (5‐HTPDC). The 5‐HT not binds to receptor, partially reuptaked by plasma membrane serotonin transporters (SERT) or degraded to 5‐hydroxyindolacetic acid (5‐HIAA) by monoamine oxidase(MAO).^138^ Rats with reduced insulin secretion have lower levels of 5‐HT and dopamine (DA). And insulin administration lowers plasma glucose levels, which increases 5‐HT levels.^139^ Synthesis and conversion of 5‐HT reduced by 44%‐71% in diabetic rats with plasma glucose levels between 500 and 600 mg%.^140^

**FIGURE 4:**
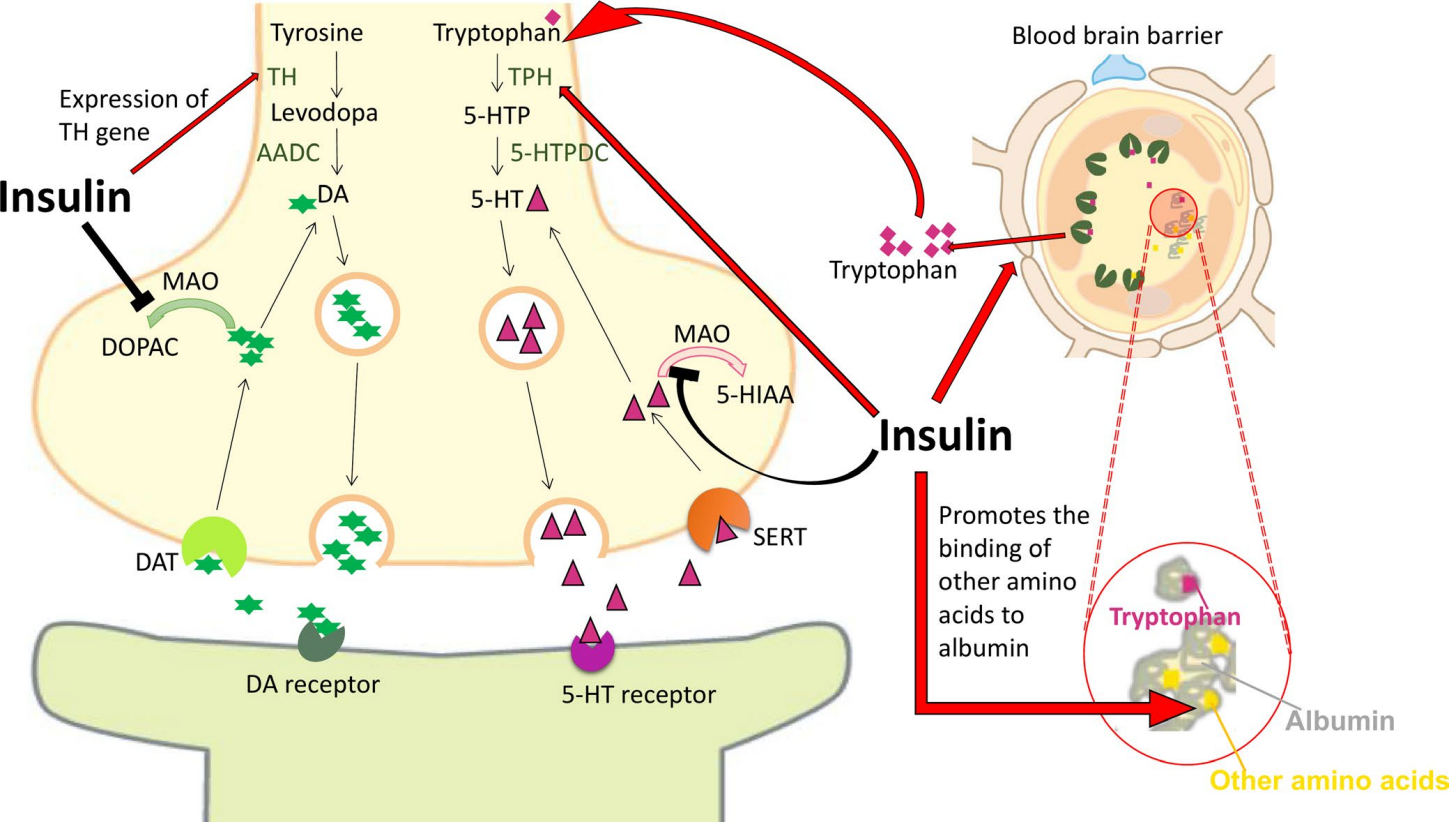
Insulin increases the supply of tryptophan to the brain. Although it promotes the binding of tryptophan to albumin, it also increases the binding of other amino acids to albumin, which increases the binding rate of tryptophan to the blood‐brain barrier transport system. Except increases tryptophan, raw material of 5‐HT synthesis, insulin also enhances the activity of 5‐HT synthetase tryptophan hydroxylase (TPH) and inhibits the activity of monoamine oxidase (MAO) which degrades 5‐HT. Insulin also activates the promoter of dopamine synthase tyrosine hydroxylase (TH), increasing the mRNA level of TH, and reduces the activity of dopamine degrading enzyme MAO, preventing the decomposition of dopamine

Insulin affects 5‐HT through a variety of pathways. First, insulin can increase the amount of 5‐HT raw material tryptophan in the brain. Injecting insulin into the periphery affects the supply of tryptophan to the brain in two opposite directions: on the one hand, insulin promotes the binding of tryptophan to albumin in the blood, reduces the amount of free tryptophan in the circulatory system, thereby reducing the transport of tryptophan to the brain; and on the other hand, insulin removes some of other amino acids from the bloodstream, which would compete with tryptophan through the shared transport system of the blood‐brain barrier, thereby increasing the influx of tryptophan into the brain. Because of these two effects, insulin increases the amount of tryptophan that enters the brain.^141^ Except increases the tryptophan intake, insulin increases the rate of neuronal serotonin synthesis.^142^ In the pineal body cultured in vitro, insulin enhances tryptophan hydroxylase activity^143^ (Figure 4).

Insulin could also contribute to the regulation of mood by influence the emotion modifier dopamine, an important monoamine neurotransmitter that is primarily responsible for delivering excitement and pleasure. One of the principles of antidepressant monoamine oxidase inhibitors is to inhibit dopamine metabolism and increase its level in brain.^144^ Destruction of the DA system is the basis for the lack of pleasure in several mental illnesses, including depression.^145^, ^146^

Tyrosine is transported to dopamine neurons by low‐ or high‐affinity amino acid transport system, then catalysed by rate‐limiting enzyme tyrosine hydroxylase (TH) in the cytoplasm and converted to levodopa. Under the catalysis of aromatic amino acid decarboxylase (AADC), the levodopa converses to dopamine and dopamine released from the monoamine transporter (VMAT) to the synaptic gap.^147^ The dopaminergic neuron terminal has dopamine transporter (DAT), an absorption device, and is a membrane protein located in the presynaptic membrane of central dopaminergic neurons. Under normal circumstances, dopamine releases into the synaptic cleft, and then DAT pumps dopamine back to the nerve endings. After part of dopamine returns to the synaptic terminals, some dopamine degraded to dihydroxyphenylacetic acid (DOPAC) in the synapse under the oxidation of MAO.^148^, ^149^

Dopamine neurons in the ventral tegmental area (VTA) and the substantia nigra pars compacta (SNc) express the insulin receptor.^150^ Experiments have shown that after peripheral injection of insulin, dopamine release increases in the nucleus accumbens, whereas in the striatum, 200,400 mU of insulin causes an increase in dopamine release, but dopamine release is inhibited in the group treated with 600 mU insulin. These data suggest that peripheral insulin injections cause changes in dopamine release in NAC and striatum.^151^ In autopsy results, decreased expression of insulin receptor‐associated signalling genes (ie, INSR, IRS1 and IRS2) resulted in genes that regulate dopamine neurotransmission in dlPFC and hippocampus (ie, DDC, TH, VMAT2, DRD1, DRD2, DRD5 and MAOB). The gene expression is reduced, which in turn leads to lower dopamine metabolism.^152^ This result is basically consistent with relevant clinical data.^150^, ^153^, ^154^

Insulin uses a biphasic manner to stimulate the cis‐regulatory element associated with the TH promoter, for instance the hypoxia‐inducible factor 1‐alpha,^155^ specific protein 1,^156^ and increases the level of TH mRNA,^157^ increases the expression of the TH gene.^154^, ^155^ Deletion of the insulin receptor on dopamine neurons reduces the rate‐limiting enzyme TH expression^158^ (Figure 4). Insulin signalling affects dopamine utilization and turnover. Insulin‐activated insulin receptors cause an increase of DA uptake by DA transporter (DAT). This process involves the PI3K/AKT signalling pathway and causes DAT to be inserted into the plasma membrane.^159^, ^160^ The level of DAT mRNA expression in theVTA/SNc of rats treated with insulin injected into the third ventricle (i.c.v.) was significantly elevated.^161^ While insulin increases dopamine uptake, its primary role in NAc and CPu is to enhance dopamine release. This dynamic regulation from dopamine of insulin release is primarily involved in the excitatory insulin‐dependent increase in striatal cholinergic interneurons (ChIs), which leads to dopamine release increased through activation of nicotinic acetylcholine (ACh) receptors (nAChRs). The effect of insulin on ChIs and DA release is mediated by the insulin receptor. In the striatum sections of food‐restricted rats, the effect of insulin on DA release was amplified, while in obese rats, the effect of insulin was diminished.^162^

In neurons, insulin is able to reduce the activity of monoamine‐degrading enzyme: MAO‐A and MAO‐B, thereby reducing monoamine clearance and increasing 5‐HT and DA signalling and prolong the monoamine half‐life after it released.^163^ Insulin receptor knockout mice have elevated levels of monoamine oxidases A and B, resulting in increased dopamine conversion and age‐related anxiety and depression‐like behaviour.^164^ However, the increase in MAO and the increase in DA metabolic breakdown caused by downregulation of insulin receptors only occurred in the striatum and NAC, which was not detected in the prefrontal cortex. This may indicate that different brain regions respond differently to insulin or insulin resistance.^164^, ^165^ MAO A and B also degrade serotonin, which affects the signal transduction of serotonin. Since serotonin signalling is also associated with depression, this pathway may also be associated with the pathogenesis of mood disorders^166^ (Figure 4).

### 3.4 Interaction of the gastrointestinal microbiome and insulin in depression

The accumulating knowledge of the correlation between gastrointestinal and depression gives rise to the microbiota‐gut‐brain (MGB) axis. The prevalence of depression in irritable bowel syndrome is reported to be 84%.^167^ In view of this situation, gut microbiota targets may be a new research direction. A few of gut microbiota genera and their metabolites can synthesize neurotransmitters and neuropeptides and essential amino acids and participate in immune and endocrine functions.^47^, ^168^, ^169^ For example, eating bifidobacterium longum could reduce anxiety in mice with colitis by improving hippocampal BDNF mRNA levels.^170^ In another study, administration of lactobacillus helveticus increased serotonin concentrations in the hippocampus and alleviated depression and anxiety.^171^, ^172^ Induction of diet‐induced obesity mice exhibits insulin resistance and a variety of depression‐like and anxiety‐like behaviours. Oral antibiotics metronidazole or vancomycin can enhance insulin signalling in the brain and relieve depression. Gut microbiota regulators could improve the balance of microorganism to ameliorate glucose metabolism defects and insulin secretion disorders by adjusting the microbiota‐gut‐brain axis. Then, gut microbiota reduces depressive behaviour through changes brain insulin signalling, inflammation, neurotransmitters and other neuroactive molecules (such as BDNF).^173^, ^174^

Gut microbes and insulin interact with each other. Increasing insulin secretion and reducing insulin resistance could improve glucose regulation, restore intestinal microbiome disorders and maintain the homeostasis of the gut microbiota.^169^, ^175^, ^176^ Under the premise that the flora remains stable, probiotics secrete a variety of signal molecules, which work through different pathways, thereby exerting antidepressant, immune regulation or regulating neurotransmission.^177^ Low levels of two major probiotic bacteria (lactic acid bacteria and bifidobacteria) are usually found in depression individuals.^178^ In the case of gut microbiota homeostasis, ingestion of lactobacillus rhamnosus increases the central mRNA expression of γ‐aminobutyric acid (GABA) receptors, while reducing depression and anxiety‐like behaviour in mice.^179^ Supplementation with lactobacillus helveticus can improve memory and cognitive ability in chronically stressed rats. This improvement in memory is associated with increased level of hippocampal norepinephrine and decreased levels of plasma corticosterone and adrenocorticotropic hormone.^171^ Previous studies have also shown that ingestion of lactobacillus helveticus can enhance memory and reduce intestinal inflammation in rats induced by neural inflammation.^178^ Understanding the interaction of the gastrointestinal microbiome with insulin provides some treatments that may be superior to standard antidepressants and shows the potential of regulating the gastrointestinal microbiome to treat depression.

## 4 CONCLUSION

The incidence of depression has gradually increased in recent years, and the effects of insulin on depression are widely studied. Therefore, it is important to clarify the potential association of depression and diabetes and explore the common physiological and pathological basis of their pathogenesis. In this article, we outlined the role and possible regulatory mechanisms of insulin, which plays an important role in the course of diabetes, in depression and depression‐like behaviour. These include the different effects and mechanisms of peripheral and intranasal insulin injections on the HPA axis, the effects of insulin on neurogenesis, synaptic plasticity and neurotrophic factors, and the positive effects of insulin on depression through monoamine neurotransmitters and gastrointestinal microbiome. Therefore, this review may provide new insight for clarifying the role of insulin on the pathogenesis of depression.

## CONFLICT OF INTEREST

The authors declare no conflict of interest.

## AUTHOR CONTRIBUTIONS

XHZ and BJL wrote the first draft of the manuscript; LHS and WY wrote sections of the manuscript; RC provided the critical revisions. All authors revised the manuscript and approved the submitted version.

## Data Availability

Data available on request.

## References

[1] Anderson RJ , Freedland KE , Clouse RE , Lustman PJ . The prevalence of comorbid depression in adults with diabetes: a meta‐analysis. Diabetes Care. 2001;24:1069‐1078. 11375373 10.2337/diacare.24.6.1069

[2] Holt RI , Sönksen PH . Growth hormone, IGF‐I and insulin and their abuse in sport. Br J Pharmacol. 2008;154:542‐556. 18376417 10.1038/bjp.2008.99 PMC2439509

[3] Greenwood EA , Pasch LA , Cedars MI , et al. Insulin resistance is associated with depression risk in polycystic ovary syndrome. Fertil Steril. 2018;110:27‐34. 29908775 10.1016/j.fertnstert.2018.03.009 PMC6392023

[4] Gulley LD , Shomaker LB , Kelly NR , et al. Indirect effects of a cognitive‐behavioral intervention on adolescent weight and insulin resistance through decreasing depression in a randomized controlled trial. J Pediatr Psychol. 2019;44(10):1163‐1173. 31393981 10.1093/jpepsy/jsz064 PMC6823102

[5] Perry BI , Khandaker GM , Marwaha S , et al. Insulin resistance and obesity, and their association with depression in relatively young people: findings from a large UK birth cohort. Psychol Med. 2020;50(4):556‐565. 30854996 10.1017/S0033291719000308 PMC7093318

[6] Rafaelsen OJ . Insulin action on the central nervous system. Acta medica Scandinavica. 1967;476:75‐84. 4865722 10.1111/j.0954-6820.1967.tb12686.x

[7] Engström W , Shokrai A , Otte K , et al. Transcriptional regulation and biological significance of the insulin like growth factor II gene. Cell Prolif. 1998;31(5–6):173‐189. 9925986 10.1111/j.1365-2184.1998.tb01196.x PMC6647699

[8] Porte D Jr , Baskin DG , Schwartz MW . Leptin and insulin action in the central nervous system. Nutr Rev. 2002;60(suppl_10):S20‐S29. 12403080 10.1301/002966402320634797

[9] Havrankova J , Schmechel D , Roth J , Brownstein M . Identification of insulin in rat brain. Proc Natl Acad Sci USA. 1978;75:5737‐5741. 364489 10.1073/pnas.75.11.5737 PMC393044

[10] Marks JL , Porte D Jr , Stahl WL , Baskin DG . Localization of insulin receptor mRNA in rat brain by in situ hybridization. Endocrinology. 1990;127:3234‐3236. 2249648 10.1210/endo-127-6-3234

[11] Chabot JG , Kar S , Quirion R . Autoradiographical and immunohistochemical analysis of receptor localization in the central nervous system. Histochem J. 1996;28:729‐745. 8968726 10.1007/BF02272147

[12] Park CR . Cognitive effects of insulin in the central nervous system. Neurosci Biobehav Rev. 2001;25:311‐323. 11445137 10.1016/s0149-7634(01)00016-1

[13] Chiu SL , Cline HT . Insulin receptor signaling in the development of neuronal structure and function. Neural Dev. 2010;5:7. 20230616 10.1186/1749-8104-5-7 PMC2843688

[14] Yokoyama K , Yamada T , Mitani H , et al. Relationship between hypothalamic‐pituitary‐adrenal axis dysregulation and insulin resistance in elderly patients with depression. Psychiatry Res. 2015;226:494‐498. 25757913 10.1016/j.psychres.2015.01.026

[15] Dean J , Keshavan M . The neurobiology of depression: An integrated view. Asian J Psychiatr. 2017;27:101‐111. 28558878 10.1016/j.ajp.2017.01.025

[16] Lim GY , Tam WW , Lu Y , Ho CS , Zhang MW , Ho RC . Prevalence of depression in the community from 30 countries between 1994 and 2014. Sci Rep. 2018;8:2861. 29434331 10.1038/s41598-018-21243-x PMC5809481

[17] Puig‐Antich J , Novacenko H , Davies M , et al Growth hormone secretion in prepubertal children with major depression. I. Final report on response to insulin‐induced hypoglycemia during a depressive episode. Arch Gen Psychiatry. 1984;41(5):455‐460. 6372735 10.1001/archpsyc.1984.01790160041004

[18] Puig‐Antich J , Novacenko H , Davies M , et al. Growth hormone secretion in prepubertal children with major depression. III. Response to insulin‐induced hypoglycemia after recovery from a depressive episode and in a drug‐free state. Arch Gen Psychiatry. 1984;41(5):471‐475. 6372736 10.1001/archpsyc.1984.01790160057006

[19] Arrieta‐Cruz I , Gutiérrez‐Juárez R . The role of insulin resistance and glucose metabolism dysregulation in the development of alzheimer´s disease. Rev Invest Clin. 2016;68:53‐58. 27103040

[20] Kumar N , Dey CS . Metformin enhances insulin signalling in insulin‐dependent and‐independent pathways in insulin resistant muscle cells. Br J Pharmacol. 2002;137:329‐336. 12237252 10.1038/sj.bjp.0704878 PMC1573500

[21] Hung YJ , Hsieh CH , Chen YJ , et al. Insulin sensitivity, proinflammatory markers and adiponectin in young males with different subtypes of depressive disorder. Clin Endocrinol (Oxf). 2007;67:784‐789. 17697007 10.1111/j.1365-2265.2007.02963.x

[22] Kern W , Peters A , Fruehwald‐Schultes B , Deininger E , Born J , Fehm HL . Improving influence of insulin on cognitive functions in humans. Neuroendocrinology. 2001;74(4):270‐280. 11598383 10.1159/000054694

[23] Marks DR , Tucker K , Cavallin MA , Mast TG , Fadool DA . Awake intranasal insulin delivery modifies protein complexes and alters memory, anxiety, and olfactory behaviors. J Neurosci. 2009;29:6734‐6751. 19458242 10.1523/JNEUROSCI.1350-09.2009 PMC2779219

[24] Reger MA , Watson GS , Frey WH III , et al. Effects of intranasal insulin on cognition in memory‐impaired older adults: modulation by APOE genotype. Neurobiol Aging. 2006;27(3):451‐458. 15964100 10.1016/j.neurobiolaging.2005.03.016

[25] Benedict C , Hallschmid M , Schmitz K , et al. Intranasal insulin improves memory in humans: superiority of insulin aspart. Neuropsychopharmacology. 2007;32:239‐243. 16936707 10.1038/sj.npp.1301193

[26] Bruggink SM , Shomaker LB , Kelly NR , et al. Insulin sensitivity, depression/anxiety, and physical fitness in at‐risk adolescents. Sports Med Int Open. 2019;3(2):E40‐E47. 31214645 10.1055/a-0889-8653 PMC6579727

[27] Hamer JA , Testani D , Mansur RB , Lee Y , Subramaniapillai M , McIntyre RS . Brain insulin resistance: a treatment target for cognitive impairment and anhedonia in depression. Exp Neurol. 2019;315:1‐8. 30695707 10.1016/j.expneurol.2019.01.016

[28] Leonard BE , Wegener G . Inflammation, insulin resistance and neuroprogression in depression. Acta Neuropsychiatr. 2020;32(1):1‐9. 31186075 10.1017/neu.2019.17

[29] Pratchayasakul W , Kerdphoo S , Petsophonsakul P , Pongchaidecha A , Chattipakorn N , Chattipakorn SC . Effects of high‐fat diet on insulin receptor function in rat hippocampus and the level of neuronal corticosterone. Life Sci. 2011;88:619‐627. 21315737 10.1016/j.lfs.2011.02.003

[30] Vogt MC , Brüning JC . CNS insulin signaling in the control of energy homeostasis and glucose metabolism ‐ from embryo to old age. Trends Endocrinol Metab. 2013;24:76‐84. 23265947 10.1016/j.tem.2012.11.004

[31] Grillo CA , Tamashiro KL , Piroli GG , et al. Lentivirus‐mediated downregulation of hypothalamic insulin receptor expression. Physiol Behav. 2007;92:691‐701. 17585961 10.1016/j.physbeh.2007.05.043 PMC2129218

[32] Passafaro M , Piëch V , Sheng M . Subunit‐specific temporal and spatial patterns of AMPA receptor exocytosis in hippocampal neurons. Nat Neurosci. 2001;4:917‐926. 11528423 10.1038/nn0901-917

[33] Oh MC , Derkach VA , Guire ES , Soderling TR . Extrasynaptic membrane trafficking regulated by GluR1 serine 845 phosphorylation primes AMPA receptors for long‐term potentiation. J Biol Chem. 2006;281:752‐758. 16272153 10.1074/jbc.M509677200

[34] Zhou W , Wang N , Yang C , Li XM , Zhou ZQ , Yang JJ . Ketamine‐induced antidepressant effects are associated with AMPA receptors‐mediated upregulation of mTOR and BDNF in rat hippocampus and prefrontal cortex. Eur Psychiatry. 2014;29:419‐423. 24321772 10.1016/j.eurpsy.2013.10.005

[35] Mizuno T , Zhang G , Takeuchi H , et al. Interferon‐gamma directly induces neurotoxicity through a neuron specific, calcium‐permeable complex of IFN‐gamma receptor and AMPA GluR1 receptor. FASEB J. 2008;22:1797‐1806. 18198214 10.1096/fj.07-099499

[36] Grillo CA , Piroli GG , Evans AN , et al. Obesity/hyperleptinemic phenotype adversely affects hippocampal plasticity: effects of dietary restriction. Physiol Behav. 2011;104:235‐241. 21036186 10.1016/j.physbeh.2010.10.020 PMC3097290

[37] Grillo CA , Piroli GG , Kaigler KF , Wilson SP , Wilson MA , Reagan LP . Downregulation of hypothalamic insulin receptor expression elicits depressive‐like behaviors in rats. Behav Brain Res. 2011;222:230‐235. 21458499 10.1016/j.bbr.2011.03.052 PMC3774048

[38] Sharma AN , Elased KM , Lucot JB . Rosiglitazone treatment reversed depression‐ but not psychosis‐like behavior of db/db diabetic mice. J Psychopharmacol. 2012;26:724‐732. 22331176 10.1177/0269881111434620

[39] Kurhe Y , Mahesh R . Pioglitazone, a PPARγ agonist rescues depression associated with obesity using chronic unpredictable mild stress model in experimental mice. Neurobiol Stress. 2016;3:114‐121. 27981184 10.1016/j.ynstr.2016.05.001 PMC5146196

[40] Evans MC , Kumar NS , Inglis MA , Anderson GM . Leptin and insulin do not exert redundant control of metabolic or emotive function via dopamine neurons. Horm Behav. 2018;106:93‐104. 30292429 10.1016/j.yhbeh.2018.10.001

[41] Steiner J , Fernandes BS , Guest PC , et al. Glucose homeostasis in major depression and schizophrenia: a comparison among drug‐naïve first‐episode patients. Eur Arch Psychiatry Clin Neurosci. 2019;269(4):373‐377. 29352386 10.1007/s00406-018-0865-7

[42] Hermanns N , Scheff C , Kulzer B , et al. Association of glucose levels and glucose variability with mood in type 1 diabetic patients. Diabetologia. 2007;50:930‐933. 17370057 10.1007/s00125-007-0643-y

[43] Young H , Benton D . The nature of the control of blood glucose in those with poorer glucose tolerance influences mood and cognition. Metab Brain Dis. 2014;29(3):721‐728. 24664179 10.1007/s11011-014-9519-2

[44] Peng Y , Liu J , Shi L , et al. Mitochondrial dysfunction precedes depression of AMPK/AKT signaling in insulin resistance induced by high glucose in primary cortical neurons. J Neurochem. 2016;137(5):701‐713. 26926143 10.1111/jnc.13563

[45] Regenold WT , Pratt M , Nekkalapu S , Shapiro PS , Kristian T , Fiskum G . Mitochondrial detachment of hexokinase 1 in mood and psychotic disorders: implications for brain energy metabolism and neurotrophic signaling. J Psychiatr Res. 2012;46:95‐104. 22018957 10.1016/j.jpsychires.2011.09.018

[46] Su L , Cai Y , Xu Y , Dutt A , Shi S , Bramon E . Cerebral metabolism in major depressive disorder: a voxel‐based meta‐analysis of positron emission tomography studies. BMC Psychiatry. 2014;14:321. 25407081 10.1186/s12888-014-0321-9 PMC4240898

[47] Vannucci SJ , Maher F , Simpson IA . Glucose transporter proteins in brain: delivery of glucose to neurons and glia. Glia. 1997;21:2‐21. 9298843 10.1002/(sici)1098-1136(199709)21:1<2::aid-glia2>3.0.co;2-c

[48] Klepper J , Engelbrecht V , Scheffer H , van der Knaap MS , Fiedler A . GLUT1 deficiency with delayed myelination responding to ketogenic diet. Pediatr Neurol. 2007;37:130‐133. 17675029 10.1016/j.pediatrneurol.2007.03.009

[49] Kahl KG , Georgi K , Bleich S , et al. Altered DNA methylation of glucose transporter 1 and glucose transporter 4 in patients with major depressive disorder. J Psychiatr Res. 2016;76:66‐73. 26919485 10.1016/j.jpsychires.2016.02.002

[50] Wilson CM , Mitsumoto Y , Maher F , Klip A . Regulation of cell surface GLUT1, GLUT3, and GLUT4 by insulin and IGF‐I in L6 myotubes. FEBS Lett. 1995;368:19‐22. 7615080 10.1016/0014-5793(95)00589-2

[51] Shimamoto S , Nakashima K , Kamimura R , et al. Insulin acutely increases glucose transporter 1 on plasma membranes and glucose uptake in an AKT‐dependent manner in chicken adipocytes. Gen Comp Endocrinol. 2019;283:113232. 31356813 10.1016/j.ygcen.2019.113232

[52] Yang K , Chen Z , Gao J , et al. The key roles of GSK‐3β in regulating mitochondrial activity. Cell Physiol Biochem. 2017;44:1445‐1459. 29190615 10.1159/000485580

[53] Zakharova IO , Sokolova TV , Bayunova LV , et al. The protective effect of insulin on rat cortical neurons in oxidative stress and its dependence on the modulation of Akt, GSK‐3beta, ERK1/2, and AMPK Activities. Int J Mol Sci. 2019;20(15):3702. 10.3390/ijms20153702 PMC6696072 31362343

[54] Plotsky PM , Owens MJ , Nemeroff CB . Psychoneuroendocrinology of depression. Hypothalamic‐pituitary‐adrenal axis. Psychiatr Clin North Am. 1998;21:293‐307. 9670227 10.1016/s0193-953x(05)70006-x

[55] Menke A . Is the HPA axis as target for depression outdated, or is there a new hope. Front Psychiatry. 2019;10:101. 30890970 10.3389/fpsyt.2019.00101 PMC6413696

[56] Board F , Wadeson R , Persky H . Depressive affect and endocrine functions; blood levels of adrenal cortex and thyroid hormones in patients suffering from depressive reactions. Archiv Neurol Psychiatry. 1957;78:612‐620. 13478217

[57] Cowen PJ . Not fade away: the HPA axis and depression. Psychol Med. 2010;40:1‐4. 19335939 10.1017/S0033291709005558

[58] Ahmad MH , Fatima M , Mondal AC . Role of hypothalamic‐pituitary‐adrenal axis, hypothalamic‐pituitary‐gonadal axis and insulin signaling in the pathophysiology of Alzheimer's disease. Neuropsychobiology. 2019;77:197‐205. 30605907 10.1159/000495521

[59] Xia L , Zhu X , Zhao Y , et al. Genome‐wide RNA sequencing analysis reveals that IGF‐2 attenuates memory decline, oxidative stress and amyloid plaques in an Alzheimer's disease mouse model (AD) by activating the PI3K/AKT/CREB signaling pathway. Int Psychogeriatr. 2019;31(07):947‐959. 10.1017/S1041610219000383 31266549

[60] Detka J , Ślusarczyk J , Kurek A , et al. Hypothalamic insulin and glucagon‐like peptide‐1 levels in an animal model of depression and their effect on corticotropin‐releasing hormone promoter gene activity in a hypothalamic cell line. Pharmacol Rep. 2019;71:338‐346. 30831439 10.1016/j.pharep.2018.11.001

[61] Guardiola‐Diaz HM , Kolinske JS , Gates LH , Seasholtz AF . Negative glucorticoid regulation of cyclic adenosine 3', 5'‐monophosphate‐stimulated corticotropin‐releasing hormone‐reporter expression in AtT‐20 cells. Mol Endocrinol. 1996;10:317‐329. 8833660 10.1210/mend.10.3.8833660

[62] Jurek B , Slattery DA , Hiraoka Y , et al. Oxytocin regulates stress‐induced Crf gene transcription through CREB‐regulated transcription coactivator 3. J Neurosci. 2015;35:12248‐12260. 26338335 10.1523/JNEUROSCI.1345-14.2015 PMC4556790

[63] Zheng WH , Quirion R . Insulin‐like growth factor‐1 (IGF‐1) induces the activation/phosphorylation of Akt kinase and cAMP response element‐binding protein (CREB) by activating different signaling pathways in PC12 cells. BMC Neurosci. 2006;7:51. 16792806 10.1186/1471-2202-7-51 PMC1534052

[64] Xiang Q , Zhang J , Li CY , et al. Insulin resistance‐induced hyperglycemia decreased the activation of Akt/CREB in hippocampus neurons: Molecular evidence for mechanism of diabetes‐induced cognitive dysfunction. Neuropeptides. 2015;54:9‐15. 26344332 10.1016/j.npep.2015.08.009

[65] Ghuman SP , Morris R , Scherzer J , et al. Neuronal responses in the brainstem and hypothalamic nuclei following insulin treatment during the late follicular phase in the ewe. Reprod Domest Anim. 2011;46:121‐129. 20403131 10.1111/j.1439-0531.2010.01605.x

[66] Bano G , Rodin DA , White A , O'Rahilly S , Nussey SS . Is the defect in pro‐hormone processing in Type 2 diabetes mellitus restricted to the beta cell. Diabet Med. 2001;18:17‐21. 11168336 10.1046/j.1464-5491.2001.00397.x

[67] Pan Y , Hong Y , Zhang QY , Kong LD . Impaired hypothalamic insulin signaling in CUMS rats: restored by icariin and fluoxetine through inhibiting CRF system. Psychoneuroendocrinology. 2013;38:122‐134. 22663897 10.1016/j.psyneuen.2012.05.007

[68] Fruehwald‐Schultes B , Kern W , Bong W , et al. Supraphysiological hyperinsulinemia acutely increases hypothalamic‐pituitary‐adrenal secretory activity in humans. J Clin Endocrinol Metab. 1999;84:3041‐3046. 10487662 10.1210/jcem.84.9.5953

[69] Tosi F , Negri C , Brun E , et al. Insulin enhances ACTH‐stimulated androgen and glucocorticoid metabolism in hyperandrogenic women. Eur J Endocrinol. 2011;164:197‐203. 21059865 10.1530/EJE-10-0782

[70] McEwen BS , Seeman T . Protective and damaging effects of mediators of stress. Elaborating and testing the concepts of allostasis and allostatic load. Ann N Y Acad Sci. 1999;896:30‐47. 10681886 10.1111/j.1749-6632.1999.tb08103.x

[71] Stranahan AM , Arumugam TV , Cutler RG , Lee K , Egan JM , Mattson MP . Diabetes impairs hippocampal function through glucocorticoid‐mediated effects on new and mature neurons. Nat Neurosci. 2008;11:309‐317. 18278039 10.1038/nn2055 PMC2927988

[72] Osmanovic J , Plaschke K , Salkovic‐Petrisic M , Grünblatt E , Riederer P , Hoyer S . Chronic exogenous corticosterone administration generates an insulin‐resistant brain state in rats. Stress. 2010;13:123‐131. 19929311 10.3109/10253890903080379

[73] Benzler J , Ganjam GK , Legler K , et al. Acute inhibition of central c‐Jun N‐terminal kinase restores hypothalamic insulin signalling and alleviates glucose intolerance in diabetic mice. J Neuroendocrinol. 2013;25:446‐454. 23301857 10.1111/jne.12018

[74] Solas M , Gerenu G , Gil‐Bea FJ , Ramírez MJ . Mineralocorticoid receptor activation induces insulin resistance through c‐Jun N‐terminal kinases in response to chronic corticosterone: cognitive implications. J Neuroendocrinol. 2013;25:350‐356. 23181759 10.1111/jne.12006

[75] van der Heide LP , Ramakers GMJ , Smidt MP . Insulin signaling in the central nervous system: Learning to survive. Prog Neurobiol. 2006;79(4):205‐221. 16916571 10.1016/j.pneurobio.2006.06.003

[76] Xia Q , Wang H , Yin H , Yang Z . Excessive corticosterone induces excitotoxicity of hippocampal neurons and sensitivity of potassium channels via insulin‐signaling pathway. Metab Brain Dis. 2019;34:119‐128. 30284676 10.1007/s11011-018-0326-z

[77] Chan O , Inouye K , Akirav E , et al. Insulin alone increases hypothalamo‐pituitary‐adrenal activity, and diabetes lowers peak stress responses. Endocrinology. 2005;146:1382‐1390. 15564337 10.1210/en.2004-0607

[78] Inouye KE , Chan O , Yue JT , et al. The effect of long‐term insulin treatment with and without antecedent hypoglycemia on neuropeptide and corticosteroid receptor expression in the brains of diabetic rats. Brain Res Bull. 2008;77:149‐157. 18672033 10.1016/j.brainresbull.2008.07.001

[79] Binder EB , Salyakina D , Lichtner P , et al. Polymorphisms in FKBP5 are associated with increased recurrence of depressive episodes and rapid response to antidepressant treatment. Nat. Genet. 2004;36:1319‐1325. 15565110 10.1038/ng1479

[80] Binder EB . The role of FKBP5, a co‐chaperone of the glucocorticoid receptor in the pathogenesis and therapy of affective and anxiety disorders. Psychoneuroendocrinology. 2009;34(Suppl 1):S186‐195. 19560279 10.1016/j.psyneuen.2009.05.021

[81] Pereira MJ , Palming J , Svensson MK , et al. FKBP5 expression in human adipose tissue increases following dexamethasone exposure and is associated with insulin resistance. Metab Clin Exp. 2014;63:1198‐1208. 24997500 10.1016/j.metabol.2014.05.015

[82] Fichna M , Krzyśko‐Pieczka I , Żurawek M , Skowrońska B , Januszkiewicz‐Lewandowska D , Fichna P . FKBP5 polymorphism is associated with insulin resistance in children and adolescents with obesity. Obes Res Clin Pract. 2018;12:62‐70. 28007534 10.1016/j.orcp.2016.11.007

[83] Bohringer A , Schwabe L , Richter S , Schachinger H . Intranasal insulin attenuates the hypothalamic‐pituitary‐adrenal axis response to psychosocial stress. Psychoneuroendocrinology. 2008;33:1394‐1400. 18804330 10.1016/j.psyneuen.2008.08.002

[84] Hallschmid M , Benedict C , Schultes B , Born J , Kern W . Obese men respond to cognitive but not to catabolic brain insulin signaling. Int J Obes (Lond). 2008;32:275‐282. 17848936 10.1038/sj.ijo.0803722

[85] Born J , Fehm HL . Hypothalamus‐pituitary‐adrenal activity during human sleep: a coordinating role for the limbic hippocampal system. Exp Clin Endocrinol Diabetes. 1998;106:153‐163. 9710353 10.1055/s-0029-1211969

[86] Benedict C , Hallschmid M , Hatke A , et al. Intranasal insulin improves memory in humans. Psychoneuroendocrinology. 2004;29:1326‐1334. 15288712 10.1016/j.psyneuen.2004.04.003

[87] Dohm FA , Beattie JA , Aibel C , Striegel‐Moore RH . Factors differentiating women and men who successfully maintain weight loss from women and men who do not. J Clin Psychol. 2001;57:105‐117. 11211279 10.1002/1097-4679(200101)57:1<105::aid-jclp11>3.0.co;2-i

[88] Clegg DJ , Riedy CA , Smith KA , Benoit SC , Woods SC . Differential sensitivity to central leptin and insulin in male and female rats. Diabetes. 2003;52:682‐687. 12606509 10.2337/diabetes.52.3.682

[89] Aleksandrova LR , Wang YT , Phillips AG . Evaluation of the Wistar‐Kyoto rat model of depression and the role of synaptic plasticity in depression and antidepressant response. Neurosci Biobehav Rev. 2019;105:1‐23. 31336112 10.1016/j.neubiorev.2019.07.007

[90] Huang CC , Lee CC , Hsu KS . The role of insulin receptor signaling in synaptic plasticity and cognitive function. Chang Gung Med J. 2010;33:115‐125. 20438663

[91] Kullmann S , Heni M , Hallschmid M , Fritsche A , Preissl H , Häring HU . Brain insulin resistance at the crossroads of metabolic and cognitive disorders in humans. Physiol Rev. 2016;96(4):1169‐1209. 27489306 10.1152/physrev.00032.2015

[92] Malenka RC , Bear MF . LTP and LTD: an embarrassment of riches. Neuron. 2004;44:5‐21. 15450156 10.1016/j.neuron.2004.09.012

[93] Zhao F , Siu JJ , Huang W , Askwith C , Cao L . Insulin modulates excitatory synaptic transmission and synaptic plasticity in the mouse hippocampus. Neuroscience. 2019;411:237‐254. 31146008 10.1016/j.neuroscience.2019.05.033 PMC6612444

[94] Liu Z , Patil IY , Jiang T , et al. High‐fat diet induces hepatic insulin resistance and impairment of synaptic plasticity. PLoS ONE. 2015;10:e0128274. 26023930 10.1371/journal.pone.0128274 PMC4449222

[95] Chiu SL , Chen CM , Cline HT . Insulin receptor signaling regulates synapse number, dendritic plasticity, and circuit function in vivo. Neuron. 2008;58:708‐719. 18549783 10.1016/j.neuron.2008.04.014 PMC3057650

[96] Pochwat B , Nowak G , Szewczyk B . An update on NMDA antagonists in depression. Expert Rev Neurother. 2019;19(11):1055‐1067. 31328587 10.1080/14737175.2019.1643237

[97] Christie JM , Wenthold RJ , Monaghan DT . Insulin causes a transient tyrosine phosphorylation of NR2A and NR2B NMDA receptor subunits in rat hippocampus. J. Neurochem. 1999;72:1523‐1528. 10098857 10.1046/j.1471-4159.1999.721523.x

[98] Jones ML , Liao GY , Malecki R , Li M , Salazar NM , Leonard JP . PI 3‐kinase and PKCζ mediate insulin‐induced potentiation of NMDA receptor currents in Xenopus oocytes. Brain Res. 2012;1432:7‐14. 22137655 10.1016/j.brainres.2011.11.020

[99] Ikonomidou C , Turski L . Why did NMDA receptor antagonists fail clinical trials for stroke and traumatic brain injury. Lancet Neurol. 2002;1:383‐386. 12849400 10.1016/s1474-4422(02)00164-3

[100] Ding S , Zhuge W , Yang J , et al. Insulin resistance disrupts the interaction between AKT and the NMDA receptor and the inactivation of the CaMKIV/CREB pathway in minimal hepatic encephalopathy. Toxicol Sci. 2017;159:290‐306. 28505381 10.1093/toxsci/kfx093

[101] Van Der Heide LP , Kamal A , Artola A , Gispen WH , Ramakers GMJ . Insulin modulates hippocampal activity‐dependent synaptic plasticity in a N‐methyl‐d‐aspartate receptor and phosphatidyl‐inositol‐3‐kinase‐dependent manner. J Neurochem. 2005;94:1158‐1166. 16092951 10.1111/j.1471-4159.2005.03269.x

[102] Costello DA , Claret M , Al‐Qassab H , et al. Brain deletion of insulin receptor substrate 2 disrupts hippocampal synaptic plasticity and metaplasticity. PLoS ONE. 2012;7:e31124. 22383997 10.1371/journal.pone.0031124 PMC3287998

[103] Cline BH , Costa‐Nunes JP , Cespuglio R , et al. Dicholine succinate, the neuronal insulin sensitizer, normalizes behavior, REM sleep, hippocampal pGSK3 beta and mRNAs of NMDA receptor subunits in mouse models of depression. Front Behav Neurosci. 2015;9:37. 25767439 10.3389/fnbeh.2015.00037 PMC4341562

[104] Kamal A , Ramakers GM , Gispen WH , Biessels GJ . Effect of chronic intracerebroventricular insulin administration in rats on the peripheral glucose metabolism and synaptic plasticity of CA1 hippocampal neurons. Brain Res. 2012;1435:99‐104. 22206925 10.1016/j.brainres.2011.11.057

[105] Recio‐Pinto E , Ishii DN . Insulin and insulinlike growth factor receptors regulating neurite formation in cultured human neuroblastoma cells. J. Neurosci. Res. 1988;19:312‐320. 3288762 10.1002/jnr.490190306

[106] Clarke DW , Boyd FT Jr , Kappy MS , Raizada MK . Insulin stimulates macromolecular synthesis in cultured glial cells from rat brain. Am J Physiol. 1985;249:C484‐489. 2415002 10.1152/ajpcell.1985.249.5.C484

[107] Fernyhough P , Mill JF , Roberts JL , Ishii DN . Stabilization of tubulin mRNAs by insulin and insulin‐like growth factor I during neurite formation. Brain Res Mol Brain Res 1989;6:109‐120. 2693875 10.1016/0169-328x(89)90044-2

[108] Baek SH , Kim EK , Goudreau JL , Lookingland KJ , Kim SW , Yu SW . Insulin withdrawal‐induced cell death in adult hippocampal neural stem cells as a model of autophagic cell death. Autophagy. 2009;5:277‐279. 19158478 10.4161/auto.5.2.7641

[109] Huang J , Wang H . Hsp83/Hsp90 physically associates with insulin receptor to promote neural stem cell reactivation. Stem Cell Reports. 2018;11:883‐896. 30245208 10.1016/j.stemcr.2018.08.014 PMC6178561

[110] Park H , Chung KM , An HK , et al. Parkin promotes mitophagic cell death in adult hippocampal neural stem cells following insulin withdrawal. Front Mol Neurosci. 2019;12:46. 30853892 10.3389/fnmol.2019.00046 PMC6395409

[111] Ha S , Ryu HY , Chung KM , Baek SH , Kim EK , Yu SW . Regulation of autophagic cell death by glycogen synthase kinase‐3β in adult hippocampal neural stem cells following insulin withdrawal. Mol Brain. 2015;8:30. 25986948 10.1186/s13041-015-0119-9 PMC4436742

[112] Chung KM , Jeong EJ , Park H , An HK , Yu SW . Mediation of autophagic cell death by type 3 Ryanodine Receptor (RyR3) in adult hippocampal neural stem cells. Front Cell Neurosci. 2016;10:116. 27199668 10.3389/fncel.2016.00116 PMC4858590

[113] Ha S , Jeong SH , Yi K , et al. Phosphorylation of p62 by AMP‐activated protein kinase mediates autophagic cell death in adult hippocampal neural stem cells. J Biol Chem. 2017;292:13795‐13808. 28655770 10.1074/jbc.M117.780874 PMC5566532

[114] Arsham AM , Neufeld TP . Thinking globally and acting locally with TOR. Curr Opin Cell Biol. 2006;18:589‐597. 17046229 10.1016/j.ceb.2006.09.005

[115] Yin XM , Ding WX , Gao W . Autophagy in the liver. Hepatology. 2008;47:1773‐1785. 18393362 10.1002/hep.22146

[116] Lim S , Rashid MA , Jang M , et al. Mitochondria‐targeted antioxidants protect pancreatic β‐cells against oxidative stress and improve insulin secretion in glucotoxicity and glucolipotoxicity. Cell Physiol Biochem. 2011;28:873‐886. 22178940 10.1159/000335802

[117] Moreira PI . Alzheimer's disease and diabetes: an integrative view of the role of mitochondria, oxidative stress, and insulin. J Alzheimers Dis. 2012;30(Suppl 2):S199‐215. 22269163 10.3233/JAD-2011-111127

[118] Bogacka I , Xie H , Bray GA , Smith SR . Pioglitazone induces mitochondrial biogenesis in human subcutaneous adipose tissue in vivo. Diabetes. 2005;54:1392‐1399. 15855325 10.2337/diabetes.54.5.1392

[119] Liu HY , Han J , Cao SY , et al. Hepatic autophagy is suppressed in the presence of insulin resistance and hyperinsulinemia: inhibition of FoxO1‐dependent expression of key autophagy genes by insulin. J Biol Chem. 2009;284:31484‐31492. 19758991 10.1074/jbc.M109.033936 PMC2781544

[120] Sun P , Ortega G , Tan Y , et al. Streptozotocin impairs proliferation and differentiation of adult hippocampal neural stem cells in vitro‐correlation with alterations in the expression of proteins associated with the insulin system. Front Aging Neurosci. 2018;10:145. 29867451 10.3389/fnagi.2018.00145 PMC5968103

[121] Dienel GA . Fueling and imaging brain activation. ASN Neuro. 2012;4:AN20120021. 10.1042/AN20120021 PMC3401074 22612861

[122] Qu ZQ , Zhou Y , Zeng YS , et al. Protective effects of a Rhodiola crenulata extract and salidroside on hippocampal neurogenesis against streptozotocin‐induced neural injury in the rat. PLoS ONE. 2012;7:e29641. 22235318 10.1371/journal.pone.0029641 PMC3250459

[123] Le Belle JE , Orozco NM , Paucar AA , et al. Proliferative neural stem cells have high endogenous ROS levels that regulate self‐renewal and neurogenesis in a PI3K/Akt‐dependant manner. Cell Stem Cell. 2011;8:59‐71. 21211782 10.1016/j.stem.2010.11.028 PMC3018289

[124] Limoli CL , Giedzinski E , Baure J , Doctrow SR , Rola R , Fike JR . Using superoxide dismutase/catalase mimetics to manipulate the redox environment of neural precursor cells. Radiat Prot Dosimetry. 2006;122:228‐236. 17166877 10.1093/rpd/ncl458

[125] Gulyaeva NV . Interplay between Brain BDNF and Glutamatergic systems: A brief state of the evidence and association with the pathogenesis of depression. Biochemistry Mosc. 2017;82:301‐307. 28320271 10.1134/S0006297917030087

[126] Tanila H . The role of BDNF in Alzheimer's disease. Neurobiol. Dis. 2017;97:114‐118. 27185594 10.1016/j.nbd.2016.05.008

[127] Huang X , Huang X , Zhou Y , et al. Association of serum BDNF levels with psychotic symptom in chronic patients with treatment‐resistant depression in a Chinese Han population. Psychiatry Res. 2017;257:279‐283. 28783576 10.1016/j.psychres.2017.07.076

[128] Takach O , Gill TB , Silverman MA . Modulation of insulin signaling rescues BDNF transport defects independent of tau in amyloid‐β oligomer‐treated hippocampal neurons. Neurobiol Aging. 2015;36(3):1378‐1382. 25543463 10.1016/j.neurobiolaging.2014.11.018

[129] Tsuneki H , Yoshida H , Endo K , et al. Different impacts of acylated and non‐acylated long‐acting insulin analogs on neural functions in vitro and in vivo. Diabetes Res Clin Pract. 2017;129:62‐72. 28511140 10.1016/j.diabres.2017.03.032

[130] Rajasekar N , Nath C , Hanif K , Shukla R . Intranasal insulin improves cerebral blood flow, Nrf‐2 expression and BDNF in STZ (ICV)‐induced memory impaired rats. Life Sci. 2017;173:1‐10. 27693383 10.1016/j.lfs.2016.09.020

[131] Shieh PB , Ghosh A . Molecular mechanisms underlying activity‐dependent regulation of BDNF expression. J Neurobiol. 1999;41:127‐134. 10504200

[132] Haas CB , Kalinine E , Zimmer ER , et al. Brain insulin administration triggers distinct cognitive and neurotrophic responses in young and aged rats. Mol Neurobiol. 2016;53:5807‐5817. 26497034 10.1007/s12035-015-9494-6

[133] Su H , Tao J , Zhang J , et al. The effects of BDNF Val66Met gene polymorphism on serum BDNF and cognitive function in methamphetamine‐dependent patients and normal controls: a case‐control study. J Clin Psychopharmacol. 2015;35:517‐524. 26280836 10.1097/JCP.0000000000000390

[134] Pavlov KA , Chistiakov DA , Chekhonin VP . Genetic determinants of aggression and impulsivity in humans. J. Appl. Genet. 2012;53:61‐82. 21994088 10.1007/s13353-011-0069-6

[135] Daily JW , Park S . Interaction of BDNF rs6265 variants and energy and protein intake in the risk for glucose intolerance and type 2 diabetes in middle‐aged adults. Nutrition. 2017;33:187‐194. 27553771 10.1016/j.nut.2016.07.001

[136] Wang Y , Zhang H , Li Y , et al. BDNF Val66Met polymorphism and plasma levels in Chinese Han population with obsessive‐compulsive disorder and generalized anxiety disorder. J Affect Disord. 2015;186:7‐12. 26209750 10.1016/j.jad.2015.07.023

[137] Lemberger L , Fuller RW , Zerbe RL . Use of specific serotonin uptake inhibitors as antidepressants. Clin Neuropharmacol. 1985;8:299‐317. 2866836 10.1097/00002826-198512000-00001

[138] Meneses A . 5‐HT system and cognition. Neurosci Biobehav Rev. 1999;23:1111‐1125. 10643820 10.1016/s0149-7634(99)00067-6

[139] Kino M , Yamato T , Aomine M . Simultaneous measurement of nitric oxide, blood glucose, and monoamines in the hippocampus of diabetic rat: an in vivo microdialysis study. Neurochem Int. 2004;44:65‐73. 12971908 10.1016/s0197-0186(03)00125-6

[140] Trulson ME , Jacoby JH , MacKenzie RG . Streptozotocin‐induced diabetes reduces brain serotonin synthesis in rats. J. Neurochem. 1986;46:1068‐1072. 2419504 10.1111/j.1471-4159.1986.tb00619.x

[141] Daniel PM , Love ER , Moorhouse SR , Pratt OE . The effect of insulin upon the influx of tryptophan into the brain of the rabbit. J Physiol. 1981;312:551‐562. 7021801 10.1113/jphysiol.1981.sp013643 PMC1275568

[142] Cangiano C , Cardelli‐Cangiano P , Cascino A , et al. On the stimulation by insulin of tryptophan transport across the blood‐brain barrier. Biochem Int. 1983;7:617‐627. 6091659

[143] Garcia RA , Afeche SC , Scialfa JH , et al. Insulin modulates norepinephrine‐mediated melatonin synthesis in cultured rat pineal gland. Life Sci. 2008;82:108‐114. 18048060 10.1016/j.lfs.2007.10.016

[144] Tekes K , Tóthfalusi L , Gaál J , Magyar K . Effect of MAO inhibitors on the uptake and metabolism of dopamine in rat and human brain. Pol J Pharmacol Pharm. 1988;40:653‐658. 3152003

[145] Grace AA . Dysregulation of the dopamine system in the pathophysiology of schizophrenia and depression. Nat Rev Neurosci. 2016;17:524‐532. 27256556 10.1038/nrn.2016.57 PMC5166560

[146] Belujon P , Grace AA . Dopamine system dysregulation in major depressive disorders. Int J Neuropsychopharmacol. 2017;20:1036‐1046. 29106542 10.1093/ijnp/pyx056 PMC5716179

[147] Berke JD . What does dopamine mean. Nat Neurosci. 2018;21:787‐793. 29760524 10.1038/s41593-018-0152-y PMC6358212

[148] Glover V , Sandler M , Owen F , Riley GJ . Dopamine is a monoamine oxidase B substrate in man. Nature. 1977;265:80‐81. 834248 10.1038/265080a0

[149] Shih JC , Chen K , Ridd MJ . Role of MAO A and B in neurotransmitter metabolism and behavior. Pol J Pharmacol. 1999;51:25‐29. 10389141

[150] Figlewicz DP . Expression of receptors for insulin and leptin in the ventral tegmental area/substantia nigra (VTA/SN) of the rat: Historical perspective. Brain Res. 2016;1645:68‐70. 26731335 10.1016/j.brainres.2015.12.041

[151] Potter GM , Moshirfar A , Castonguay TW . Insulin affects dopamine overflow in the nucleus accumbens and the striatum. Physiol Behav. 1999;65:811‐816. 10073485 10.1016/s0031-9384(98)00233-9

[152] Mansur RB , Fries GR , Subramaniapillai M , et al. Expression of dopamine signaling genes in the post‐mortem brain of individuals with mental illnesses is moderated by body mass index and mediated by insulin signaling genes. J Psychiatr Res. 2018;107:128‐135. 30391805 10.1016/j.jpsychires.2018.10.020 PMC6278951

[153] Caravaggio F , Hahn M , Nakajima S , Gerretsen P , Remington G , Graff‐Guerrero A . Reduced insulin‐receptor mediated modulation of striatal dopamine release by basal insulin as a possible contributing factor to hyperdopaminergia in schizophrenia. Med Hypotheses. 2015;85:391‐396. 26118462 10.1016/j.mehy.2015.06.011 PMC5323257

[154] Senthilkumaran M , Johnson ME , Bobrovskaya L . The effects of insulin‐induced hypoglycaemia on tyrosine hydroxylase phosphorylation in rat brain and adrenal gland. Neurochem Res. 2016;41:1612‐1624. 26935743 10.1007/s11064-016-1875-3

[155] Fiory F , Mirra P , Nigro C , et al. Role of the HIF‐1α/Nur77 axis in the regulation of the tyrosine hydroxylase expression by insulin in PC12 cells. J Cell Physiol. 2019;234:11861‐11870. 30536670 10.1002/jcp.27898

[156] Nakashima A , Ota A , Sabban EL . Interactions between Egr1 and AP1 factors in regulation of tyrosine hydroxylase transcription. Brain Res Mol Brain Res. 2003;112:61‐69. 12670703 10.1016/s0169-328x(03)00047-0

[157] Vietor I , Rusnak M , Viskupic E , Blazicek P , Sabban EL , Kvetnansky R . Glucoprivation by insulin leads to trans‐synaptic increase in rat adrenal tyrosine hydroxylase mRNA levels. Eur J Pharmacol. 1996;313:119‐127. 8905338 10.1016/0014-2999(96)00508-0

[158] Könner AC , Hess S , Tovar S , et al. Role for insulin signaling in catecholaminergic neurons in control of energy homeostasis. Cell Metab. 2011;13:720‐728. 21641553 10.1016/j.cmet.2011.03.021

[159] Carvelli L , Morón JA , Kahlig KM , et al. PI 3‐kinase regulation of dopamine uptake. J Neurochem. 2002;81:859‐869. 12065645 10.1046/j.1471-4159.2002.00892.x

[160] Lin Z , Zhang PW , Zhu X , et al. Phosphatidylinositol 3‐kinase, protein kinase C, and MEK1/2 kinase regulation of dopamine transporters (DAT) require N‐terminal DAT phosphoacceptor sites. J Biol Chem. 2003;278:20162‐20170. 12660249 10.1074/jbc.M209584200

[161] Figlewicz DP , Szot P , Chavez M , Woods SC , Veith RC . Intraventricular insulin increases dopamine transporter mRNA in rat VTA/substantia nigra. Brain Res. 1994;644:331‐334. 8050044 10.1016/0006-8993(94)91698-5

[162] Stouffer MA , Woods CA , Patel JC , et al. Insulin enhances striatal dopamine release by activating cholinergic interneurons and thereby signals reward. Nat Commun. 2015;6:8543. 26503322 10.1038/ncomms9543 PMC4624275

[163] Gupta D , Kurhe Y , Radhakrishnan M . Antidepressant effects of insulin in streptozotocin induced diabetic mice: modulation of brain serotonin system. Physiol Behav. 2014;129:73‐78. 24582678 10.1016/j.physbeh.2014.02.036

[164] Kleinridders A , Cai W , Cappellucci L , et al. Insulin resistance in brain alters dopamine turnover and causes behavioral disorders. Proc Natl Acad Sci USA. 2015;112:3463‐3468. 25733901 10.1073/pnas.1500877112 PMC4371978

[165] Herculano‐Houzel S . The glia/neuron ratio: how it varies uniformly across brain structures and species and what that means for brain physiology and evolution. Glia. 2014;62:1377‐1391. 24807023 10.1002/glia.22683

[166] Bortolato M , Chen K , Shih JC . Monoamine oxidase inactivation: from pathophysiology to therapeutics. Adv Drug Deliv Rev. 2008;60:1527‐1533. 18652859 10.1016/j.addr.2008.06.002 PMC2630537

[167] Banerjee A , Sarkhel S , Sarkar R , Dhali GK . Anxiety and Depression in Irritable Bowel Syndrome. Indian J Psychol Med. 2017;39:741‐745. 29284804 10.4103/IJPSYM.IJPSYM_46_17 PMC5733421

[168] Rieder R , Wisniewski PJ , Alderman BL , Campbell SC . Microbes and mental health: A review. Brain Behav Immun. 2017;66:9‐17. 28131791 10.1016/j.bbi.2017.01.016

[169] Simpson CA , Mu A , Haslam N , Schwartz OS , Simmons JG . Feeling down? A systematic review of the gut microbiota in anxiety/depression and irritable bowel syndrome. J Affect Disord. 2020;266:429‐446. 32056910 10.1016/j.jad.2020.01.124

[170] Bercik P , Park AJ , Sinclair D , et al. The anxiolytic effect of Bifidobacterium longum NCC3001 involves vagal pathways for gut‐brain communication. Neurogastroenterol Motil. 2011;23:1132‐1139. 21988661 10.1111/j.1365-2982.2011.01796.x PMC3413724

[171] Liang S , Wang T , Hu X , et al. Administration of Lactobacillus helveticus NS8 improves behavioral, cognitive, and biochemical aberrations caused by chronic restraint stress. Neuroscience. 2015;310:561‐577. 26408987 10.1016/j.neuroscience.2015.09.033

[172] Morshedi M , Valenlia KB , Hosseinifard ES , et al. Beneficial psychological effects of novel psychobiotics in diabetic rats: the interaction among the gut, blood and amygdala. J Nutr Biochem. 2018;57:145‐152. 29730508 10.1016/j.jnutbio.2018.03.022

[173] Kreznar JH , Keller MP , Traeger LL , et al. Host genotype and gut microbiome modulate insulin secretion and diet‐induced metabolic phenotypes. Cell Rep. 2017;18:1739‐1750. 28199845 10.1016/j.celrep.2017.01.062 PMC5325228

[174] Soto M , Herzog C , Pacheco JA , et al. Gut microbiota modulate neurobehavior through changes in brain insulin sensitivity and metabolism. Mol Psychiatry. 2018;23:2287‐2301. 29910467 10.1038/s41380-018-0086-5 PMC6294739

[175] Wehmeier UF , Piepersberg W . Biotechnology and molecular biology of the alpha‐glucosidase inhibitor acarbose. Appl Microbiol Biotechnol. 2004;63:613‐625. 14669056 10.1007/s00253-003-1477-2

[176] Mahana D , Trent CM , Kurtz ZD , et al. Antibiotic perturbation of the murine gut microbiome enhances the adiposity, insulin resistance, and liver disease associated with high‐fat diet. Genome Med. 2016;8:48. 27124954 10.1186/s13073-016-0297-9 PMC4847194

[177] Sanada K , Nakajima S , Kurokawa S , et al Gut microbiota and major depressive disorder: a systematic review and meta‐analysis. J Affect Disord. 2020;266:1‐13. 32056863 10.1016/j.jad.2020.01.102

[178] Luo J , Wang T , Liang S , Hu X , Li W , Jin F . Ingestion of Lactobacillus strain reduces anxiety and improves cognitive function in the hyperammonemia rat. Sci China Life Sci. 2014;57:327‐335. 24554471 10.1007/s11427-014-4615-4

[179] Bravo JA , Forsythe P , Chew MV , et al. Ingestion of Lactobacillus strain regulates emotional behavior and central GABA receptor expression in a mouse via the vagus nerve. Proc. Natl Acad Sci USA. 2011;108:16050‐16055. 21876150 10.1073/pnas.1102999108 PMC3179073

